# Increasing the robustness of uplift modeling using additional splits and diversified leaf select

**DOI:** 10.1057/s41270-022-00186-3

**Published:** 2022-10-12

**Authors:** Frank Oechsle

**Affiliations:** Karlsruhe Institute of Technology (KIT), 76131 Karlsruhe, Germany

**Keywords:** Churn, Prevention, Uplift modeling, Local errors, Decision trees, Additional splits

## Abstract

While the COVID-19 pandemic negatively affects the world economy in general, the crisis accelerates concurrently the rapidly growing subscription business and online purchases. This provokes a steadily increasing demand of reliable measures to prevent customer churn which unchanged is not covered. The research analyses how preventive uplift modeling approaches based on decision trees can be modified. Thereby, it aims to reduce the risk of churn increases in scenarios with systematically occurring local estimation errors. Additionally, it compares several novel spatial distance and churn likelihood respecting selection methods applied on a real-world dataset. In conclusion, it is a procedure with incorporated additional and engineered decision tree splits that dominates the results of an appropriate Monte Carlo simulation. This newly introduced method lowers probability and negative impacts of counterproductive churn prevention campaigns without substantial loss of expected churn likelihood reduction effected by those same campaigns.

## Introduction

Pejić Bach et al. (2021, p. 1) define churn as “a situation when customer stops buying products or using services from a company.” Regarding the telecommunication industry, as an industry that for a long time proactively handles churn  (Hashmi et al. 2013), they correspondingly describe that “churn management aims to minimize the churn using various retention strategies to prevent customers from canceling subscriptions, such as offering new devices or services.” Irrespective of the field, one can differentiate between the two churn management disciplines prevention and retention depending on the moment of churn announcement. Prevention combines churn avoiding measures that take place before the customer announces churn while retention means the bunch of actions in the period between churn announcement and expiration of the contract. Companies naturally want preferably narrow churn funnels, which first of all is less churn announcements and therefore less churn. Thus, a critical factor for success in the upcoming (subscription) business era will be a strong churn management, as far as possible in a preventive way.

However, in practice, there is still no trusted concept of reducing churn in a preventive measure. That applies to uplift techniques, which are comparing the customers responses depending on the inclusion in a churn prevention campaign and all the more to response modeling. One reason is the rarity of the event churn in comparison with, e.g., purchase, which complicates its prediction. Another challenging aspect is that failures tend to generate additional churn (Radcliffe 2007b). Failures mean false selections in terms of customers would not have churned if they would not have received emails or any other contacting. This results in at least futile churn prevention efforts (Ascarza 2018).

The paper counteracts those momentous misjudgments of probabilities with a diversifying portfolio approach. This concept by dint of additional and engineered decision tree splits trades in expected churn probability for distance in the feature space. Simultaneously, it is able to reduce the risk of churn increasing churn prevention campaigns considerably in a setting with systematically assumed local estimation errors.

The fundamental idea of the line of thought is the true lift model of Lo (2002), which considers the incremental impact of an action toward the target variable, in this case churn, as the guide for decision-making. In order to train a decision tree to estimate churn probability increments as defined by Lo, the paper uses and adapts the real-world dataset of Kevin Hillstrom (2008) provided in *The minethatdata email analytics and data mining challenge*. Hence, it obtains a partition of the feature space in which it randomly incorporates the local errors mentioned above in a next step. Finally, it exercises different campaign-selection methods within the framework of a Monte Carlo simulation. The results of this simulation demonstrate the superiority of the portfolio approach in a scenario as described, notably in comparison with the classic approach.

## Related work

The prediction of uplifts as per Lo is theoretically clear and sufficiently comprehensible (Radcliffe 2007b; Kane et al. 2014; Guelman et al. 2015). However, with a few mixed exceptions (Manahan 2005; Radcliffe 2007b; Devriendt et al. 2021), empirical results as well as best practices and track records in business are not existing in the churn context.

Concerning this matter, Diemert et al. (2018, 2021) quote missing publicly available real-world datasets as a fundamental problem for the research on successful usage of uplift models (UM) in general and moreover provide a very large dataset (25M rows, 12 features). Additionally, they mention Hillstroms dataset as “the second largest and most popular uplift prediction dataset” (Diemert et al. 2018, p. 3) and note that ”in the field of UM, a notable exception to private datasets is the Hillstrom study (64,000 samples) collecting the sales results of an email marketing campaign from the 2000s” (Diemert et al. 2021, p. 2). This research will base the simulations on this exact Hillstrom dataset in the remainder of the paper.

Radcliffe (2007b, p. 13) uses the same line when he says “performance of uplift models on fabricated test data is often a particularly unreliable indicator of likely performance on real-world data. A significant challenge is therefore to find suitable data that can be made publicly available for benchmarking.” Not related to this, he brings up that “in practice, most of the real difficulties with uplift modeling derive from noise” (Radcliffe 2007b, p. 13). He describes several reasons for this noise (addition of estimation errors while fitting a difference, considerably unbalanced treated and control population, uplift phenomenon way smaller than absolute outcome rates) and states “a wide variety of methods to control noise, including careful variable selection and binning methodologies, bagging, stratified sampling, and k-way cross-validation methods” (Radcliffe 2007b, p. 13).

Shaar et al. (2016) underline Radcliffe’s perception with their statements “uplift models show high sensitivity to noise and disturbance, which leads to unreliable results” (Shaar et al. 2016, p. 1) and “most of real-world datasets contains noise and disturbances, specially for uplift modeling, as uplift effects tend to be smaller than the real treatment effect” (Shaar et al. 2016, p. 9). They allow for that with their disturbance effects minimizing approach called Pessimistic Uplift Modeling. Furthermore, they show among others using Hillstroms dataset “that our approach outperforms the existing approaches, especially in the case of high noise data environment” (Shaar et al. 2016, p. 1). Their procedure is geared to Lai (2006), who wants to maximize the probability that customers belong to the group that shows the desired response when treated or that does not show the desired response when not treated. Furthermore, it supplements Lai’s method with weights representing the predicted cases proportions of the whole population. Thus, Shaar et al. (2016) generate additional certainty on the expected outcomes by incorporating the overall frequency of an event.

The latest research toward uplift modeling mainly focuses on noise, disturbance, uncertainty, and estimation errors (Athey et al. 2015; Lo and Pachamanova 2015; Oechsle et al. 2016; Athey and Imbens 2016; Zhao et al. 2017; Rößler et al. 2021). Summing up Zhao et al. (2017, p. 8) put it in a nutshell while describing that their contribution is in a first step to “present a way to obtain an unbiased estimate of the expected response under an uplift model which has not been available in the literature.”

Whereas aforesaid papers attend to the uplift modeling challenges from a technical and engineering emphasis, Oechsle and Schönleber (2020) examine the problem of unreliable expected outcomes to a greater extent from a business perspective, in this case churn business. They “investigate the effect of suddenly upcoming estimation errors due to moving environments in the subscription business” (Oechsle and Schönleber 2020, p. 3). As a moving environment, they subsume “dynamic surrounding parameters” like “company-intern changes such as mandatory price increases, product migrations owing to technical improvement, tariff launches of competitors, or other specific events influencing customer groups in undetermined ways” (Oechsle and Schönleber 2020, p. 3). They suppose those “game-changing events” to systematically generate estimation errors, which in the uplift and churn context can be very disadvantageous, exceedingly when similar customers, that is local neighbors in the feature space, are selected. Concretely, they define circles with radius *R* around random error seeds *E* and attribute the users (or customers) *U* with Euclidean distance *r* to *E* an unnoticed change in expected uplift $\Delta$ to $\Delta '$ appropriate to

1 $$\begin{aligned} \Delta ' = {\left\{ \begin{array}{ll} \Delta &{} r > R, \\ \Delta \left[ 1 - 2\cos \left( \frac{\pi r}{2 R}\right) \right] , &{} r \le R \end{array}\right. } \end{aligned}$$

Finally, they indicate supported by simulations that it can be beneficial in defective scenarios to use distance regarding customer selection techniques.

The idea of locally occurrent unanticipated changes in churn probabilities is supported by several publications concerning the topic of churn in the neighborhood of influential churners (Dasgupta et al. 2008; Kusuma et al. 2013; Droftina et al. 2015a, b). For example, Droftina et al. (2015b, p. 1) assert that “highly influential customers deserve special attention, since their churns can also trigger churns of their peers.” Correspondingly, Kusuma et al. (2013) show on a real-world dataset that when 50 percent of the peers of users yet churned, those users’ churn rate is two times the overall churn rate among all users.

This paper picks up the idea of noise and uncertainty typified by spatially specified sources of error and exert it on a real-world dataset (Hillstrom), which previously is tailored to a churn scenario. A decision tree is trained on that dataset and it is acted upon the splitting/pruning via novel selection methods targeted to a predefined churn prevention campaign. The introduced methods are meant to regard distance in the feature space, which is well able to be done per decision tree. Besides that established decision trees employed for uplift modeling only use differences of probabilities for splitting, that is particularly they disregard distances, nor do they use pruning (Rzepakowski and Jaroszewicz 2010). Thus, common decision trees, as well as various other procedures, have an issue with locally occurring errors. The research randomly incorporates these errors in a concluding Monte Carlo Simulation (MC) which “is a very useful mathematical technique for analyzing uncertain scenarios and providing probabilistic analysis of different situations” (Raychaudhuri 2008, p. 9) while “the basic principle for applying MC analysis is simple and easy to grasp” (Raychaudhuri 2008, p. 9). It thereby provides evidence for the superiority of its approach. Certainly, even an perfectly engineered prediction model experiences problems if the described errors arise after a perfect estimation process. Hence, the focus is not to derive the most accurate prediction model, in this case, the most sophisticated decision tree, but rather to reliably implement an arbitrary proper decision tree for using the novel selection methods. In the following third chapter, the methodology will be described in depth.

The contribution of the research therefore consists of a) a publicly available uplift analysis on a real-world dataset and b) a straight forward feasible and nevertheless promising approach for daily practice c) based on decision trees combined with a distance respecting course of action d) in the rarely considered and eminently fraught with risk uplift modeling field churn, which intensifies some of the general problems uplift modeling have to deal with.

## Methodology

As seen in the comparing work of Zhao et al. (2017), Oechsle and Schönleber (2020), or Radcliffe and Surry (2011), the direct path is the superior one of the two popular uplift modeling approaches (direct uplift modeling versus two separate models subtracted afterward). Thus, let there be a decision tree with $I \in {\mathbb {N}}$ leaves for the direct estimation of the uplift

2 $$\begin{aligned} \Delta = p_0 - p_1, \end{aligned}$$

of a churn prevention campaign whereas $p_0$ , respectively, and $p_1$ display the churn probability without, respectively, and with treatment. Let further $\Delta _i$ for $i = 1,2,\ldots ,I$ be the (correctly) estimated and therefore expected uplift for the customers enclosed in leaf *i*, whereas w.l.o.g. for simplification, only positive uplifts $\Delta _i$ are assumed. Leaves with estimated negative uplifts would be excluded from the first for every respectable churn prevention campaign. Let, in addition, $C_i$ be the center of the leaf *i* consisting of the average values of all features across the customers of the leaf *i*. Then the distance $d_{ij}$ of two leaves *i* and *j* pursuant to an arbitrary metric, e.g., Minkowski, is defined as the distance of their centers $C_i$ and $C_j$ appropriate to this very metric.

Also let the best leaf *b* be defined as the leaf with the highest dedicated uplift

3 $$\begin{aligned} \Delta _b = \max _{i=1,\ldots ,I} \Delta _i \end{aligned}$$

and the contained customers equivalently stand for the best customers in the same vein.

Typically for a churn prevention campaign, as well as for every other uplift campaign, the best customers are selected as far as the allocated budget allows it. That is one ignores distances and absolutely concentrates on uplifts.

However, the paper presents selection methods (*best k*, *max dist*, *tradeoff*, and *add split*), which take account of distances as well. Some of them are recent (*best k* for $k>3$ and especially *add split*), while some of them were already introduced by Oechsle and Schönleber (2020). The subsequent listing defines them and distinguishes the classic selection method.

Classic: Selects all the customers in the best leaf *b* and thus focuses on uplift.
Best k: Randomly selects 1/*k* of the customers in the *k* best leaves and thus trades off uplift against diversification.
Max dist: Randomly selects half of the customers in the best leaf *b*, and half of the customers in the leaf *i* where the distance to leaf *b* is maximal. Thus, it focuses on distance.
Tradeoff: Randomly selects half of the customers in the best leaf *b*, and half of the customers in the leaf *t* which is defined via
  4 $$\begin{aligned} \Delta _t = \min _{i=1,\ldots ,I} \frac{\Delta _b-\Delta _i}{d_{bi}} \end{aligned}$$
  Thus it considers likewise distance and uplift.
add split: synthetically conducts an additional split in the best leaf *b* just as in the second best leaf, which, respectively, bisect the corresponding leaves concerning the quantity of customers. That is it selects half of the customers in the best leaf and half of the customers in the second best leaf with the pairwise highest distance. Thus, it considers likewise distance and uplift.

## Numerical evaluation

*The minethatdata email analytics and data mining challenge* of Kevin Hillstrom (2008) marks the starting basis for our research. It is inspired by Diemert et al. (2018, 2021), Shaar et al. (2016) and the winning entry of Radcliffe (2008), who approached the exercise via uplift modeling. His underlying thoughts, independent of the won competition, are illustrated in a separate paper (Radcliffe 2007a), albeit he zooms in on sales instead of churn.

Hillstroms dataset includes the results of an email marketing campaign relating to the customer behavior in terms of website visits and purchasing. More precisely, it contains 64.000 customers who last purchased within twelve months and afterward were involved in an email test (2/3 were randomly chosen to receive an email campaign featuring merchandise, 1/3 were randomly chosen to not receive an email campaign). During a period of two weeks following the email campaign, anew purchases were tracked.

Therefore, in the following research, *Churn* is defined as *did not buy again in a certain period of time*, which is represented by the binary target variable *conversion*. Its two possible values, 1 for *customer purchased again within two weeks after the email campaign took place* and 0 for *customer did not purchase again within two weeks after the email campaign took place*, provide a churn prediction target as per definition of Pejić Bach et al. (2021) introduced in the first chapter. 578 out of Hillstroms 64.000 customers purchased again within the above-mentioned two weeks. This is a conversion rate of 0.9% which fits to the rareness of the prediction target in ordinary churn prevention cases.

Against this background, a decision tree has been developed on Hillstrom’s dataset. Preparative tasks have been a) engineering of features to result in only dealing with numeric input variables (seven features), b) calculation of z-Scores for standardization of the predictors, and c) explicit exclusion of the information whether a customer was targeted by the email campaign or not. Finally, the tree itself was built on a 80/20 training/validation split of the sample.

There is no more model-tuning since the research does not seek for the best predicting model but one reasonable partitioning of the feature space into leaves in order to utilize the selection methods specific to decision trees.

So the feature space of Hillstroms dataset was sectioned into subareas: the leaves of the decision tree. Every single customer, also the 20% in the validation subset, could be assigned to its corresponding leaf. Casually spoken the whole dataset was scored with the on itself derived model. For this purpose, the relative frequency of the value 1 of the binary target variable among the customers of the dedicated leaf defines the estimation of the conversion probability per leaf, respectively, its customers. Vice versa, the complementary probability represents the likelihood of the above-defined event churn according to the customers in that specific leaf.

To obtain the basic framework for the hereinafter described simulations, the differentiation between the customers that received an email and those who did not preliminary was performed. That is the conversion or rather the churn probability grouped by email recipients and non-email recipients was computed per leaf. By subtraction of the churn probability with email from the churn probability without email, $\Delta$ [cf. Eq. (2)] was generated as the real and correctly estimated effect of the churn prevention campaign per customer, in the absence of noise and uncertainty typified by spatially specified sources of error. For the generation of these errors, the simulations adapt the concept of Oechsle and Schönleber (2020), which was previously outlined and discussed [cf. Eq. (1)].

As described above based on Hillstroms dataset, a decision tree is engineered, which complies with the requirements of the methodology introduced in the third section. Concretely, the tree consists of $I=9$ leaves with $\Delta _i > 0$ for $i = 1,2,\ldots ,9$, whereas the uplifts represent the reduction in likelihood of churn (did not buy again) due to the email campaign in Hillstroms scenario. The chosen metric is the Euclidean distance.

In the following passage, the selection methods as listed in Sect. 3 are compared by a Monte Carlo simulation predicated on the described decision tree. An additional construction detail is the stipulated minimal leaf size of 4800 customers, which represents 7.5% of the whole dataset and, respectively, 9.4% of the training dataset. The reason is that this is an in practice imaginable campaign size and the quantitative comparability of the leaf sizes supports the elucidated selection methods.

Eight miscellaneous radii are used for the construction of the circularly occurring errors [cf. (1)] as listed in Table 2. The error radius *R* ranges from zero to two times $d_{\varnothing C_{b}}$, which is defined as the average Euclidean distance per customer to the center $C_b$ of the best leaf *b*. While $R=0$ serves as a baseline without failures, $R=2d_{\varnothing C_{b}}$ somehow will mark a break even point when it comes to the economic logic of the prevention campaign.

**Table 1:** Quintessence of runs with $R > 0$

Selection method	$\varnothing {\mathbb {E}}[\Delta ]$	$\varnothing$# Failures	$\varnothing {\mathbb {E}}[\Delta ]$ Failures
Classic	0.008	181.7	− 0.0039
Best 2	0.0063	178.1	− 0.0028
Best 3	0.0058	190.1	− 0.0024
Max dist	0.0044	164.9	− 0.0017
Tradeoff	0.0063	178.9	− 0.0029
Add split	0.0069	143.4	− 0.0022

**Fig. 1:**
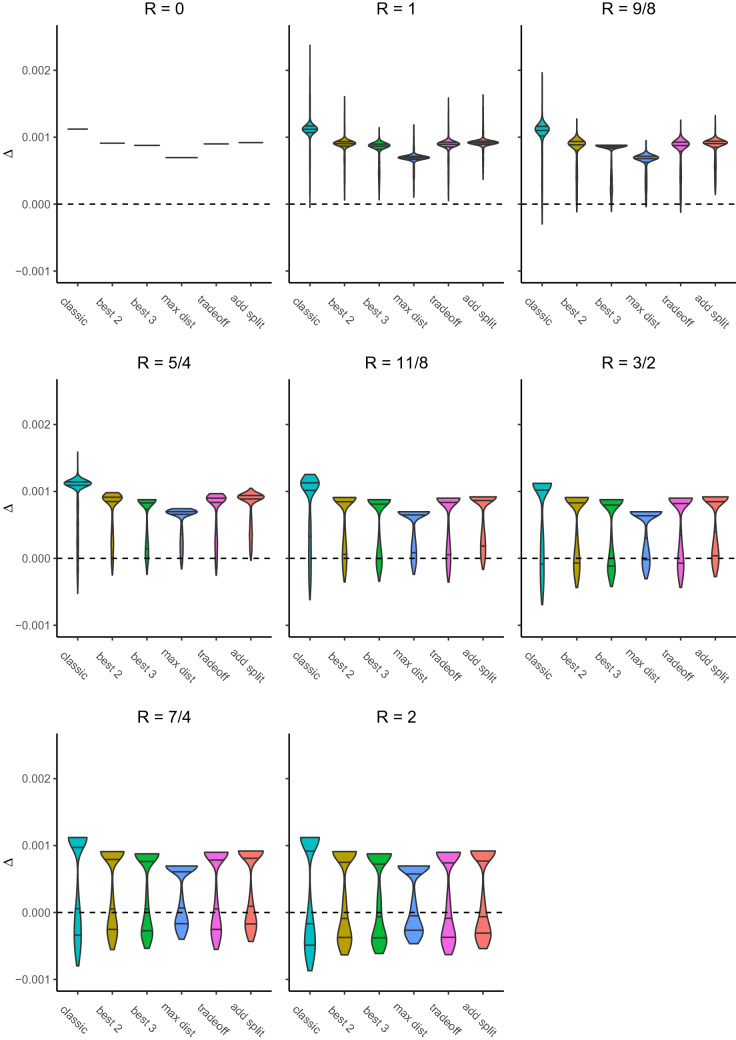
Comparison of average uplift per selected customer for different selection methods and radii *R* (in units of $d_{\varnothing C_{b}}$)

The research performs 1000 runs per error radius and with it benchmarks six selection methods (classic, best k for $k = 2$, best k for $k = 3$, max dist, tradeoff, add split) by means of the expected $\Delta$ values per customer. The underlying decision tree is always the same, while the position of the error seed *E* randomly alters. Figure 1 visualizes the statistical distributions of the results, explicitly the distributions of the achieved average uplift per selected customer and per employed selection method. Table 1 depicts the averages per selection method (for the eight times 1000 runs) of achieved (and therefore expected) uplift, number of failures and achieved uplift among failures. More precisely ${\mathbb {E}}[\Delta ]$ specifies the average (per 1000 runs) carried out average uplift per selected customer. The runs among the each undertaken 1000 runs overall that produce negative average uplifts per customer are counted as failures. Vice versa the complementary runs are counted as successes, which later will be relevant for the reading of Table 2. ${\mathbb {E}}[\Delta ]$Successes and ${\mathbb {E}}[\Delta ]$Failures consequently denote the respective average of the average uplifts generated by the dedicated successes and accordingly failures.

**Table 2:** Summary of simulation results

$R/d_{\varnothing C_{b}}$	Selection method	${\mathbb {E}}[\Delta ]$	# Successes	${\mathbb {E}}[\Delta ]$ Successes	# Failures	${\mathbb {E}}[\Delta ]$ Failures	Campaign size
0	Classic	0.012	1000	0.012	0	–	6055
0	Best 2	0.01	1000	0.01	0	–	6008
0	Best 3	0.008	1000	0.008	0	–	6964
0	Max dist	0.006	1000	0.006	0	–	6894
0	Tradeoff	0.01	1000	0.01	0	–	5965
0	Add split	0.01	1000	0.01	0	–	6084
1	Classic	0.012	999	0.012	1	$-$0.001	6055
1	Best 2	0.009	1000	0.009	0	–	6008
1	Best 3	0.007	1000	0.007	0	–	6964
1	Max dist	0.006	1000	0.006	0	–	6894
1	Tradeoff	0.009	1000	0.009	0	–	5965
1	Add split	0.01	1000	0.01	0	–	6084
9/8	Classic	0.011	981	0.011	19	$-$0.001	6055
9/8	Best 2	0.008	991	0.008	9	$-$0.001	6008
9/8	Best 3	0.007	991	0.007	9	0.00	6964
9/8	Max dist	0.006	997	0.006	3	0.00	6894
9/8	Tradeoff	0.008	991	0.008	9	$-$0.001	5965
9/8	Add split	0.009	1000	0.009	0	–	6084
5/4	Classic	0.009	928	0.01	72	$-$0.002	6055
5/4	Best 2	0.007	935	0.008	65	$-$0.001	6008
5/4	Best 3	0.006	925	0.007	75	$-$0.001	6964
5/4	Max dist	0.005	955	0.005	45	$-$0.001	6894
5/4	Tradeoff	0.007	932	0.008	68	$-$0.001	5965
5/4	Add split	0.008	999	0.008	1	0.00	6084
11/8	Classic	0.008	862	0.01	138	$-$0.003	6055
11/8	Best 2	0.007	859	0.008	141	$-$0.001	6008
11/8	Best 3	0.005	822	0.007	178	$-$0.001	6964
11/8	Max dist	0.005	877	0.005	123	$-$0.001	6894
11/8	Tradeoff	0.007	858	0.008	142	$-$0.002	5965
11/8	Add split	0.007	937	0.008	63	$-$0.001	6084
3/2	Classic	0.007	790	0.01	210	$-$0.003	6055
3/2	Best 2	0.006	770	0.008	230	$-$0.002	6008
3/2	Best 3	0.005	724	0.007	276	$-$0.002	6964
3/2	Max dist	0.004	798	0.005	202	$-$0.001	6894
3/2	Tradeoff	0.006	766	0.008	234	$-$0.002	5965
3/2	Add split	0.006	842	0.008	158	$-$0.001	6084
7/4	Classic	0.005	637	0.01	363	$-$0.004	6055
7/4	Best 2	0.004	635	0.009	365	$-$0.003	6008
7/4	Best 3	0.004	629	0.007	371	$-$0.003	6964
7/4	Max dist	0.003	650	0.006	350	$-$0.002	6894
7/4	Tradeoff	0.004	635	0.009	365	$-$0.003	5965
7/4	Add split	0.005	655	0.008	345	$-$0.002	6084
2	Classic	0.004	531	0.011	469	$-$0.005	6055
2	Best 2	0.003	563	0.009	437	$-$0.004	6008
2	Best 3	0.003	578	0.007	422	$-$0.003	6964
2	Max dist	0.002	569	0.006	431	$-$0.002	6894
2	Tradeoff	0.003	566	0.009	434	$-$0.004	5965
2	Add split	0.003	563	0.009	437	$-$0.003	6084

In Fig. 1 it is very striking that the classic selection approach comes along with the highest level of uncertainty. That is the results of the classic selection method are furthest spread as measured by values of $\Delta$. Conversely the alternative methods, second to none *best 3*, generate more dense ranges of outcomes. Particularly, as consolidated can be seen in Table 1, in comparison the classic approach not only most frequently (separate from *best 3*) led to failures, namely negative values of average uplift per customer ($\Delta$), but also induced clearly more grave failures, viz., lowest values of ${\mathbb {E}}[\Delta ]$Failures. This circumstance becomes even more apparent in Table 2 whose composition will be explained below.

Table 2 consists of 48 rows (eight radii times six selection methods), which, respectively, represent the results of the according unique radius and selection method combination in the above described each 1000 runs. To that effect, columns one and two identify the radii (as a multiple of $d_{\varnothing C_{b}}$) and the selection methods. ${\mathbb {E}}[\Delta ]$, ${\mathbb {E}}[\Delta ]$Successes and ${\mathbb {E}}[\Delta ]$Failures, and therefore columns three to seven, have already been explained with Table 1. Concluding the column, campaign size contains the number of contacted customers per selection method, which, due to the simulation construction, does not vary within the different runs. The analysis controls for this dimension to ensure comparability of the selection methods.

In the first column, as previously mentioned, the error radius varies from $R=0$ to $R=2d_{\varnothing C_{b}}$. While $R=0$ constitutes a perfect surrounding with no need to deviate from the classic proceeding, $R=2d_{\varnothing C_{b}}$ delivers failures with nearly every second run (469 out of 1000 for the classic method) and thus contests the general idea of preventing churn.

In-between these boundaries, the superiority of the classic approach becomes apparent in terms of ${\mathbb {E}}[\Delta ]$. But it is also the approach with the permanently lowest ${\mathbb {E}}[\Delta ]$ Failures and an oftentimes highest number of failures. The alternative selection methods lower these effects. By doing so, the add split approach is most suitable since it creates considerably the fewest failures. Additionally, these few failures come along with the highest ${\mathbb {E}}[\Delta ]$ Failures. Above all, the add split selection demands the lowest risk premium (as measured by ${\mathbb {E}}[\Delta ]$) for the gained robustness in results. In the case of $R/d_{\varnothing C_{b}}=7/4$ even none.

## Conclusion and discussion

The research described in this paper illustrates well-known challenges with churn prevention campaigns on a real-world dataset. It shows with the help of the previously churn-tailored Hillstrom dataset that noise and uncertainty represented by local spatial errors pose a veritable problem, which can economically destroy whole churn campaigns, especially with the classic selection approach. Thereby, it naturally plays a decisive role how voluminous relevant arising errors are. Lastly, it is demonstrated that there exist distance respecting alternative selection methods that largely give better results, dependent on the emergence of errors in terms of error radius *R*.

The most remarkable insight finally came from the *add split* selection. This method synthetically conducts additional splits in the best leaves before it selects the customers in the thereby arising subareas with the pairwise highest Euclidean distance. It directly influences the generation of the decision tree itself, because depending on the interpretation of the dodge, it either steps in the splitting rules or it intervenes in the pruning of the tree. By all means, the *add split* selection method revealed the most promising results. That implies that there are situations in which it can be beneficial to diverge from common ways of decision tree construction by, for example, adding supposedly (by the textbook) needless splits. By departing from the concept of expected values, this strategy evidently helps reducing abortive churn prevention campaigns.

In less risky scenarios ($R/d_{\varnothing C_{b}}\le 1$), there is no reason for not choosing the classic selection approach. However, in error-prone settings ($R/d_{\varnothing C_{b}}>1$), distance respecting selection approaches based on decision trees are able to outperform the classic way. This appears in the reduced number of churn increasing churn prevention campaigns, as well as in the reduced extent of failures. In only slightly more inconvenient settings ($9/8\le R/d_{\varnothing C_{b}}\le 5/4$), it is possible to reduce failures by switching from the classic method, respectively, even to avoid failures completely by using selection method *add split*. In clearly more inconvenient settings ($11/8\le R/d_{\varnothing C_{b}}\le 3/2$) solely *add split* yields a respectable reduction to an acceptable level of uncertainty. In adverse surroundings ($R/d_{\varnothing C_{b}}\ge 7/4$), the distance-based methods again outperform the classic approach. Only the rationale of the campaign on the whole is questioned by a failure quota of 1/3 to 1/2.

In an overall view, the findings can lead to feasible concepts for uplift modeling in general and especially in the churn prevention context, which will be of highest interest for the in all likelihood still growing subscription economy and the e-commerce business. At this juncture, the methodology equipes each technically correct evolved decision tree with more reliability in practical applications and thus is a valuable tool for every practitioner.
